# Human astrocytes: structure and functions in the healthy brain

**DOI:** 10.1007/s00429-017-1383-5

**Published:** 2017-03-09

**Authors:** Flora Vasile, Elena Dossi, Nathalie Rouach

**Affiliations:** Neuroglial Interactions in Cerebral Physiopathology, Center for Interdisciplinary Research in Biology, Collège de France, CNRS UMR 7241, INSERM U1050, Labex Memolife, PSL Research University, Paris, France

**Keywords:** Astrocytes, Human, Brain, Physiology, Neuroglial interactions

## Abstract

Data collected on astrocytes’ physiology in the rodent have placed them as key regulators of synaptic, neuronal, network, and cognitive functions. While these findings proved highly valuable for our awareness and appreciation of non-neuronal cell significance in brain physiology, early structural and phylogenic investigations of human astrocytes hinted at potentially different astrocytic properties. This idea sparked interest to replicate rodent-based studies on human samples, which have revealed an analogous but enhanced involvement of astrocytes in neuronal function of the human brain. Such evidence pointed to a central role of human astrocytes in sustaining more complex information processing. Here, we review the current state of our knowledge of human astrocytes regarding their structure, gene profile, and functions, highlighting the differences with rodent astrocytes. This recent insight is essential for assessment of the relevance of findings using animal models and for comprehending the functional significance of species-specific properties of astrocytes. Moreover, since dysfunctional astrocytes have been described in many brain disorders, a more thorough understanding of human-specific astrocytic properties is crucial for better-adapted translational applications.

## Introduction

Since the early discovery that the range of astroglial functions extends considerably beyond passive structural support, astrocytes have cemented their position as crucial determinants of proper neuronal function. Astrocytes are now considered as full-fledged participants in brain circuitry and processing, and display a large spectrum of functions at the cell level, such as the formation, maturation and elimination of synapses, ionic homeostasis, clearance of neurotransmitters, regulation of extracellular space volume, and modulation of synaptic activity and plasticity (Araque et al. 2014; Dallérac and Rouach 2016). It has additionally been demonstrated that they are involved in rhythm generation and neuronal network patterns (Fellin 2009; Lee et al. 2014; Poskanzer and Yuste 2016). More broadly, it is important to assess the inter-species relevance of this set of findings, keeping in mind the ultimate goal of translational applications. While animal models—mostly rodents—have been critical for determining the aforementioned astrocytic properties, early evidence has led to the hypothesis that their functions may differ somewhat: first, the astrocyte-to-neuron ratio increases with the evolutionary stage of a given species (Bass et al. 1971; Leuba and Garey 1989) and, second, the structure, morphology and diversity of human astrocytes differ greatly from that of rodents (Andriezen 1893). The incredible variety of processes in which astrocytes are involved suggests that a change in their characteristics will alter their contribution to neuronal functions. In this context, it is of crucial importance to define the scope in which data gathered using the rodent animal model might be pertinently transposed to the human species. Limited access to healthy human tissue evidently creates an obstacle to the investigation of human astrocytes, but foetal samples, healthy brain tissue resected from surgical procedures, and post-mortem samples have allowed the study of astrocytic properties in the human brain. Here, we review findings concerning the structure, gene profile and function of human astrocytes, and how they differ from those of rodents.

## Astrocyte-to-neuron ratio in evolution

Arguably the most intuitive evidence that glia might play a particularly important role in the cognitive functions exhibited by humans is a correlation between the brain glia-to-neuron ratio and the state of evolution of species (Han et al. 2013). On one hand, *C. elegans* possess 302 neurons but only 56 glial cells, a ratio of 0.18 (Oikonomou and Shaham 2011), and the rat cerebral cortex contains a mean glia/neuron ratio of 0.4 (Bass et al. 1971). On the other hand, the whole human adult brain was found to contain a one-to-one ratio (Azevedo et al. 2009) and the human cerebral cortex a ratio of 1.4 (Friede 1954; Pelvig et al. 2008). Additionally, comparison of glia-to-neuron ratio in different human cortical areas with that of macaques and chimpanzees also displayed a significant augmentation of the ratio in humans (Sherwood et al. 2006). Herculano-Houzel (2014) provides a comprehensive account of glia-to-neuron ratios across species, and its significance. A major caveat to the phylogenetic theory of glia-to-neuron ratio increase with brain evolution is the report that large mammals show a pronounced increase of glia proportion in the brain, to wit 80% for African savannah elephants (Goodman et al. 2009) and whales (Hawkins and Olszewski 1957). While some argue that the increase in glial number in the human brain does not ensure higher cognitive ability, but merely provide enhanced metabolic support required by the higher energetic demands that stem from larger neurons and brain size (Hawkins and Olszewski 1957), it appears that bigger neurons do not require more energy (Herculano-Houzel 2011). Rather, the correlation between glial number and neuronal density might account for varying glia-to-neuron ratios across species (Herculano-Houzel 2014). Corroborating this idea, it appears that the said ratio might not be uniform in the brain, and even within cortical areas: indeed, the glia-to-neuron index was reported to only reach 0.23 in the cerebellum (against 1.4 in the cerebral cortex) (Azevedo et al. 2009), and the human visual cortex areas 17 and 18 were found to display ratios of 0.6 and 1, respectively (Leuba and Garey 1989). This was posited to be a consequence of varying neuronal densities across structures, and might not be specific to the human brain (Herculano-Houzel 2014). While these studies provide invaluable insight into the cell type composition of the human brain, they mostly fail to distinguish glial classes. Strikingly Pelvig and colleagues report a glial population composed of 75% oligodendrocytes, 20% astrocytes and 5% microglia in the human cerebral cortex (Pelvig et al. 2008). However, this is not in agreement with a study in the Rhesus monkey cerebral cortex which displayed a distribution of 57% astrocytes, 35% oligodendrocytes and 7% microglial cells (Peters et al. 1991).

## Complex structure and morphology of human astroglia

**Fig. 1:**
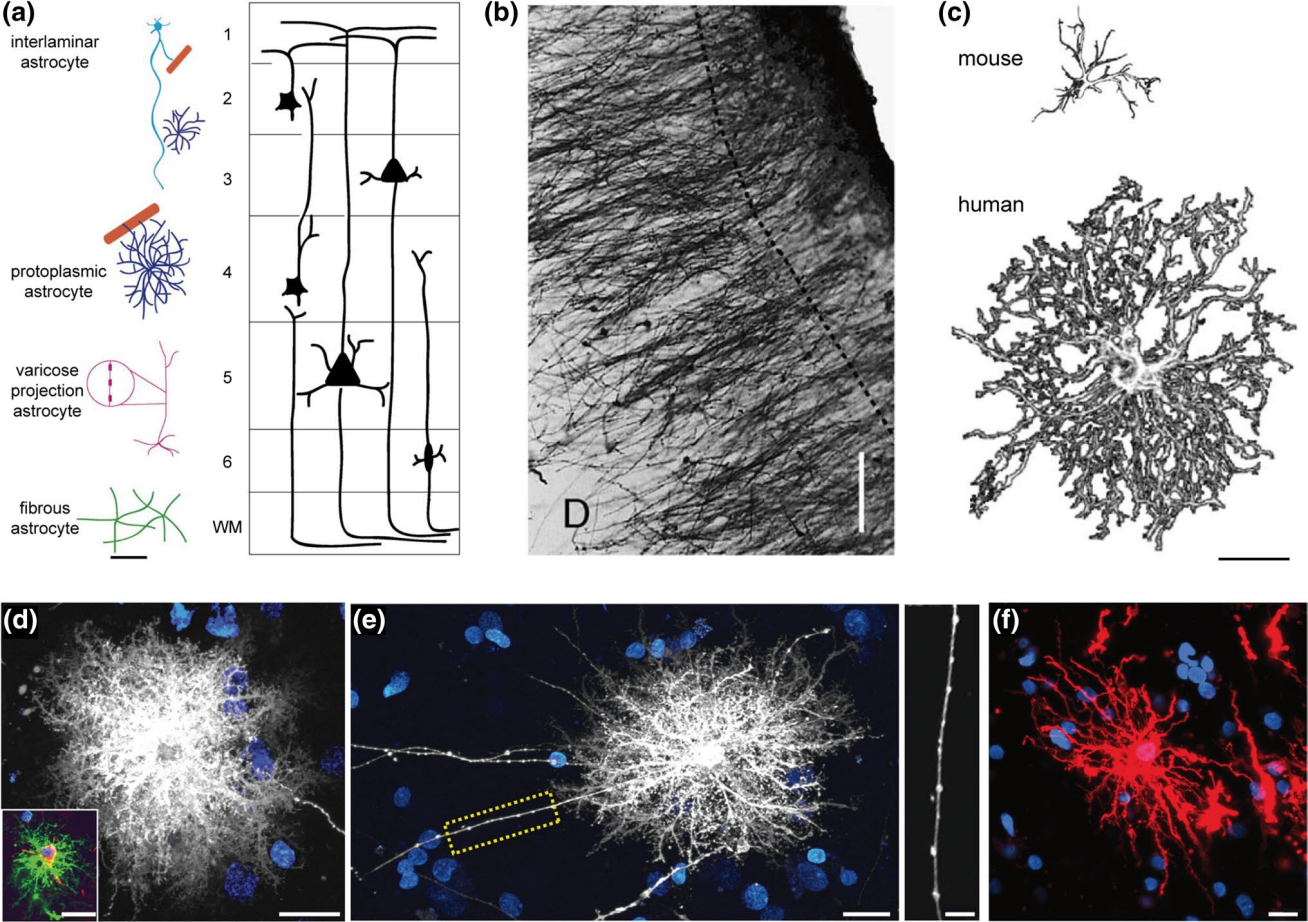
Distinct classes of astrocytes in the human brain. **a** Location of the four distinct types of human astrocytes within different layers of the cortex. *Scale bar* 100 µm. **b** Interlaminar astrocytes in the human occipital cerebral cortex. The *dashed line* indicates the limit of layer I. *Scale bar* 100 µm. **c** *Graphical representation* of mouse (*top*) and human (*bottom*) cortical protoplasmic astrocytes, showing that human astrocytes are almost threefold larger and more symmetrical than mouse astrocytes. *Scale bar* 25 µm. **d** Representative human protoplasmic astrocyte diolistically labelled. *Inset* colocalization with GFAP (*green*). *Scale bars* 20 µm. **e** Representative varicose projection astrocyte (*left*) and *enlarged view* of the area indicated by the *yellow box*, which highlights the varicosities along the processes. *Scale bars left*, 20 µm; *right*, 10 µm. **f** Representative human fibrous astrocyte. *Scale bar* 20 µm. [**a, c** adapted from (Oberheim et al. 2006); **b** from (Colombo and Reisin 2004); **d**–**f** adapted from (Oberheim et al. 2009)]

Although the implications of a higher glia-to-neuron ratio remain elusive, the structural and morphological properties of human astrocytes have been characterised as clearly different from those of evolutionarily lower mammals such as the mouse. In a pioneering study led in 1893 at University College London, Andriezen and Lond already grasped the complexity of human astrocyte morphology, employing the Golgi staining method to identify distinct groups: the ‘caudate neuroglial fibre’, the ‘stellate fibre cell’ and the ‘protoplasmic neuroglia cell’ (Andriezen 1893). Other pioneering neuroscientists have contributed to the characterisation of human astrocytes in the early days of neurobiology (Martinotti 1889; Retzius 1894; Cajal 1897), but although the evolutionary change of neuronal features were thoroughly studied (Nimchinsky et al. 1999; Yáñez et al. 2005; Prabhakar et al. 2006), data regarding human astrocytes remained sparse. Around the turn of the century, Colombo and colleagues drew attention to interlaminar astroglia—referred to as caudate neuroglial fibres by Andriezen and Lond (1893)—a primate-specific type of astrocytes. Relying on recent advances in imaging technology, Oberheim and associates later provided a more comprehensive account of human astrocytes classes, structures and morphology by studying post-mortem human tissues: human astrocytes are characterised by a heavy expression of glial fibrillary acidic protein (GFAP) that increases with age (Nichols et al. 1993). Furthermore, four classes of structurally and anatomically distinct GFAP^+^ astrocytes exist in the human brain (Fig. 1a): interlaminar, located in layers I and II; protoplasmic in layers III and IV; varicose projections in layers V and VI; and fibrous astroglia in the white matter. Each class is described below.

### Interlaminar astroglia

Layer I of the cortex was found to play host to primate-specific type of astrocyte: the interlaminar astroglia (Fig. 1b). These cells appear to have two types of processes: tangential fibres travelling radially; and ‘cable-like’, long, vertical astroglial processes (Colombo and Reisin 2004). Further characterisation of the latter by Colombo and colleagues showed millimetre-long, tortuous and varicosity-free interlaminar projections (Korzhevskii et al. 2005; Oberheim et al. 2009) originating from the somata located in the neuronal cell body-free layer I, and terminating in layer III or IV cortical neuropil in large buttons. Electron microscopy studies revealed that these endings possessed a multilamellar structure and mitochondria (Colombo et al. 1997). Furthermore, these interlaminar astroglial processes develop postnatally in the cerebral cortex of Ceboidea monkeys and humans, rather than originating from radial glial cells (Colombo et al. 1997). Despite interlaminar astrocytes being primate-specific, (Oberheim et al. 2009) noted differences between those found in primate and in human brains. Human interlaminar astrocytes are more numerous, and they display small round cell bodies, in contrast with the oblong somata found in their primate counterpart. An additional notable difference is the presence, in humans, of short processes that extend in all directions and participate in the pial glia limitans by forming a network of GFAP fibres. The functional significance of this interlaminar astroglia is yet to be determined, but it has been posited by Oberheim and colleagues that its features hint at a potential role in long-distance intra-cortical communication and coordination. The fact that disruption of intralaminar processes has been observed in pathological states associated with neuronal loss, such as Down’s syndrome (Colombo et al. 2005) and Alzheimer’s disease (Colombo et al. 2002), suggests a certain importance for neuronal support.

### Protoplasmic astroglia

The protoplasmic astrocyte (Fig. 1c, d) is the most abundant astrocyte type in the human brain, as well as in the primate and rodent brains. The complexity of human protoplasmic astrocytes, however, distinguishes them from the corresponding cells in other mammals. Oberheim and colleagues (2009) observed them to be located in layers II to VI of the cerebral cortex, and although the cell body size of human protoplasmic astrocytes equates that of rodents, their processes are notably longer (97.9 µm against 37.2 µm in rodents). Moreover, they extend an average of 37.5 GFAP^+^ processes in uniformly distributed manner while those of rodents only send 3.75 main GFAP^+^ processes that can be polarised. These increases in processes length and number lead to a remarkable enhancement of the domain of the protoplasmic astrocyte, which presents an average diameter of 142.6 µm against 56 µm in rodents (Oberheim et al. 2009). Diolistic labeling of processes in combination with GFAP staining next allowed Oberheim et al. (2009) to observe fine processes and characterise them as bulbous. Additionally, labelling of two adjacent protoplasmic astrocytes with lipophilic dyes enabled them to conclude that, as in rodents, they are organised into domains. Nonetheless, human domains showed more extended overlap with an average area of 204.7 µm^2^ and a summated length of overlapping processes of 53 µm against 11.8 µm^2^ and 4.2 µm in rodents, respectively. This differential overlap is justified by the enhanced size of human astrocytes. The manner in which human protoplasmic astrocytes cover synapses and the vasculature also appears to be at variance with rodent ones: in humans and rodents alike, nearly all protoplasmic astrocytes endfeet contact blood vessels, but in the human variety, GFAP completely covers them, as opposed to the rosette-like coverage observed in rodents. Finally, given that synaptic density is comparable in human and rodent brains (1397 vs. 1100 million synapses per mm^3^), that the volume of human protoplasmic astrocytes is increased by a factor of 16.5 compared to their rodent counterparts, and that a domain of rodent astrocyte can cover between 20,000 and 120,000 synapses, Oberheim and colleagues estimated that a single human protoplasmic astrocyte might ensure the coverage of between 270,000 and 2 million synapses. Human protoplasmic astrocytes might, therefore, have the potential to modulate inter-neuronal communication of, and locally integrate information from an amazingly large number of synapses, providing exceptional computational power.

### Varicose projection astroglia

A third type of GFAP^+^ astrocyte present in the human brain described by Oberheim et al. (2009) is the varicose projection astrocyte (Fig. 1e). Specific to humans and higher-order primates, they sparsely reside in layers V–VI and are characterised by long fibres containing varicosities distributed around 10 µm apart. The main GFAP^+^ processes are straighter and less branched, potentially indicating that they make fewer synaptic contacts than protoplasmic astrocytes do. Varicose projection astrocytes send between one and five long - up to 1 mm - processes terminating in the neuropil or on blood vessels, through either direct contact with vasculature walls or en passant contact with capillaries. Fine processes revealed with DiI-based diolistic labelling show a spiny and sharp appearance, as opposed to the bulbous processes observed in protoplasmic astrocytes (Oberheim et al. 2009). The fact that varicose projection astrocytes were only reported in human and higher-order primates, and that those found in chimpanzees were seen to be smaller and less complex than those in humans suggests a particular importance of this astrocyte type in human-specific cognitive functions. However, their precise significance is still unknown. It has been proposed that the varicosities ensure compartmentalisation of the subcellular sections along the process, and that long processes might secure long-distance communication across cortical layers, similar to interlaminar astrocytes, and even between grey and white matter (Oberheim et al. 2009).

### Fibrous astroglia

Deeper in the cerebral cortex, in the white matter, can be found fibrous astroglia (Fig. 1f). This last type of GFAP^+^ cell is little differentiated between primate and non-primate brains, but human fibrous astrocytes have been found to be markedly larger than rodent ones (183.2 vs. 85.6 µm in diameter). They are distinguishable from protoplasmic astrocytes thanks to their larger size, less branched and straighter processes, and fewer fine processes (Oberheim et al. 2009). Although they do not display a domain organisation, they appear to be equally distant from one-another, a characteristic attributed by the authors to the fact that they provide support for the axon tract. A role for fibrous astrocytes in metabolic support seems clear, as most contact the vasculature, but given the absence of synapses in the white matter, they most likely do not modulate neuronal activity.

## Gene expression profile of human astroglia

While there have been accounts of human astrocyte structure and morphology in the past century, studies of gene expression patterns to infer functional properties of human astrocytes proved to be a greater challenge, for methodological reasons. Until recently, techniques to acutely purify astrocytes lacked physiological salience as they necessitated culturing nervous tissue in serum, which is not part of astrocytes’ environment in physiology and consequently induces reactive changes (Zamanian et al. 2012). Zhang et al. (2016) circumvented these limitations by developing a technique to acutely purify human foetal and adult astrocytes using immunopanning (Zhang et al. 2016). Antibodies targeted against HepaCam, a human astrocyte surface protein, allowed obtaining purified cultures of primary human astrocytes and subsequently a transcriptome database. Transcriptomic analysis revealed that a large number of genes that were classically highly expressed in rodent astrocytes, such as GFAP, ALDH1L1, GLUL, AQP4, SLC1A2, SLC1A3 and GJB6, were also enriched in human astrocytes. Interestingly, only 30% of human astrocyte-enriched genes were also enriched in mice and, conversely, 52% of mouse astrocyte-enriched genes were also enriched in human astrocytes. This is supported by transcriptome analysis indicating that glial transcripts show the greatest differences in gene expressions between human and mice (Miller et al. 2010) and that astrocytes-associated genes are less preserved than neuron-associated genes throughout evolution (Hawrylycz et al. 2015). Similarly, this implies putative functional differences between rodent and human brains; investigating the functional properties of proteins coded by the genes specifically enriched in human astrocytes could, therefore, give insight into the specific functional properties of human astrocytes.

Significantly, at least three genes coding for proteins involved in Ca^2+^ signalling were found to be enriched in human compared to mouse astrocytes: RYR3, MRVI1 and RGN. RYR3 encodes ryanodine receptor type 3, a member of ryanodine receptors (RyRs), which are intracellular Ca^2+^-permeable channels expressed on the sarcoplasmic and endoplasmic reticula. They are activated by Ca^2+^ entry through plasma membrane Ca^2+^ channels and induce a massive and rapid Ca^2+^ release from intracellular stores (Zalk et al. 2015). RyR3 is mostly expressed in the brain (Hakamata et al. 1992), but RyR2 is the most widely expressed type of RyR in the mammalian brain (McPherson and Campbell 1993; Giannini et al. 1995). One could posit that expression of RYR3 in human astrocytes might allow enhanced or more refined Ca^2+^ signalling. However, properties of the different RyR isoforms are difficult to compare owing to the different experimental conditions used to study them. RyR2 and RyR3 conductances for Ca^2+^ and K^+^ were seen to be similar (Carney et al. 2010). The difference might be a consequence of either the quantity of RyRs and/or their specific location within the astrocyte. Intringuingly, both proteins encoded by MRVI1 and RGN inhibit Ca^2+^ signalling and intracellular accumulation. The few studies on the MRVI1 protein, also referred to as IRAG, show that in smooth muscle cells, IRAG interacts with inositol triphosphate receptors (IP3Rs) (Ammendola et al. 2001) and inhibits IP3-induced Ca^2+^ release through a NO/PRKG1-dependent mechanism (Fritsch et al. 2004). IRAG could, therefore, ensure refinement and enhanced temporal resolution of Ca^2+^ signalling through the negative regulation of IP3-mediated release from intracellular stores. Regucalcin, encoded by RGN, is a Ca^2+^-binding protein that has been shown to be expressed in rat brain neurons; it is involved in Ca^2+^ homeostasis through regulation of Ca^2+^-ATPase activity to prevent Ca^2+^ accumulation in microsomes and resulting toxicity (Yamaguchi 2012). This regulation was reported to be decreased in aged rats (Yamaguchi 2012), and could, therefore, at least in neurons, be implicated in the cognitive decline associated with ageing. Accordingly, increased expression in human astrocytes compared to both mouse astrocytes and human neurons points towards a particular importance of tight regulation of Ca^2+^ homeostasis in these cells.

Second, a collection of genes involved in metabolism were found to be highly expressed in human compared to mouse astrocytes: APOC2 encodes apolypoprotein C-2, which participates in fatty acid metabolism by activating the enzyme lipoprotein lipase that hydrolyses triglycerides to provide free fatty acids for cells (Kim et al. 2006). Additionally, AMY2B—also enriched in human astrocytes—codes for amylase alpha 2B, a secreted protein that hydrolyses 1,4-alpha-glucoside bonds in oligosaccharides and polysaccharides, and thus allows the catalysis of the first step in the digestion of dietary starch and glycogen (Omichi and Hase 1993). Notably, in a study comparing gene expression profile of human brain with that of different non-human primates (Cáceres et al. 2003), an upregulation of CA2, expressed in glia and involved in the generation and transport of lactate to neurons for energy supply (Deitmer 2002) was observed in human samples. Drawing on a comparison of metabolic rate data between humans and macaques, the authors proposed that metabolism rate might be unusually high in human brains. It is worth noting that this does not appear to be a result of an increased brain size, since it has been observed that larger brains tend to have lower metabolic rates per unit tissue (Aiello and Wheeler 1995). Consequently, high metabolic rate can be expected to support the complex computation underlying the higher cognitive abilities of humans.

This transcriptomal analysis also discloses information on the developmental regulation of cellular processes and gene expression in human astrocytes. First, it has been shown that astrocyte precursor cells (APCs) had a particularly proliferative phenotype, whereas astrocytes obtained from postnatal tissue resulted to be mature non-proliferative cells. On the one hand, APCs highly expressed a number of proliferative genes such as MKI67 and TOP2A, genes involved in mitosis (e.g. TPX2, NUSAP1), cell cycle (e.g. E2F5), differentiation (e.g. HES6) and migration (e.g. TNC). On the other hand, there was a marked increase in the expression of gap junction genes GJA1 and GJB6, Wnt pathway inhibitory factor WIF1, genes involved in immunity (TLR4), Ca^2+^ signalling (RYR3), inhibitory neurotransmission (GABRA2), and neurotransmitter recycling (GLUL) in mature astrocytes compared to APCs (Zhang et al. 2016). To the best of our knowledge, an equivalent study on the gene expression developmental regulation has not been carried out in rodents; it, therefore, remains unclear whether this is specific to humans. Finally, a set of genes did not possess mouse orthologues but were found to be enriched in astrocytes compared to other cells types. It is unclear what functions their encoded proteins bare, but it is to be considered that they might contribute to the enhanced functions of human astrocytes compared to their mouse counterpart.

An independent microarray study on post-mortem tissue samples from humans, chimpanzees, rhesus macaques and pigtail macaques uncovered a six-fold and two-fold increase in expression of THBS4 and THBS2 mRNA, respectively, in human brain samples compared to non-human primate samples (Cáceres et al. 2007). Noteworthy, while the existence and direction of change in expression of abundant transcripts can be reliably monitored by microarray studies, quantification of absolute expression levels and detection of weakly expressed genes are challenging using this method (Draghici et al. 2006). Nonetheless, THBS4 and THBS2 genes code for thrombospondin proteins, extracellular-matrix glycoproteins secreted by astrocytes and involved in synaptogenesis and neurite growth (Christopherson et al. 2005), and have been reported to be involved in cortical synaptic remodelling and plasticity after injury (Eroglu et al. 2009). Accordingly, thrombospondins secreted by astrocytes could be responsible for a modification of synapse number/organisation/dynamics and embody an evolutionary change of the human brain.

## Functional properties of human astroglia

### Neuronal survival and synaptogenesis.

**Fig. 2:**
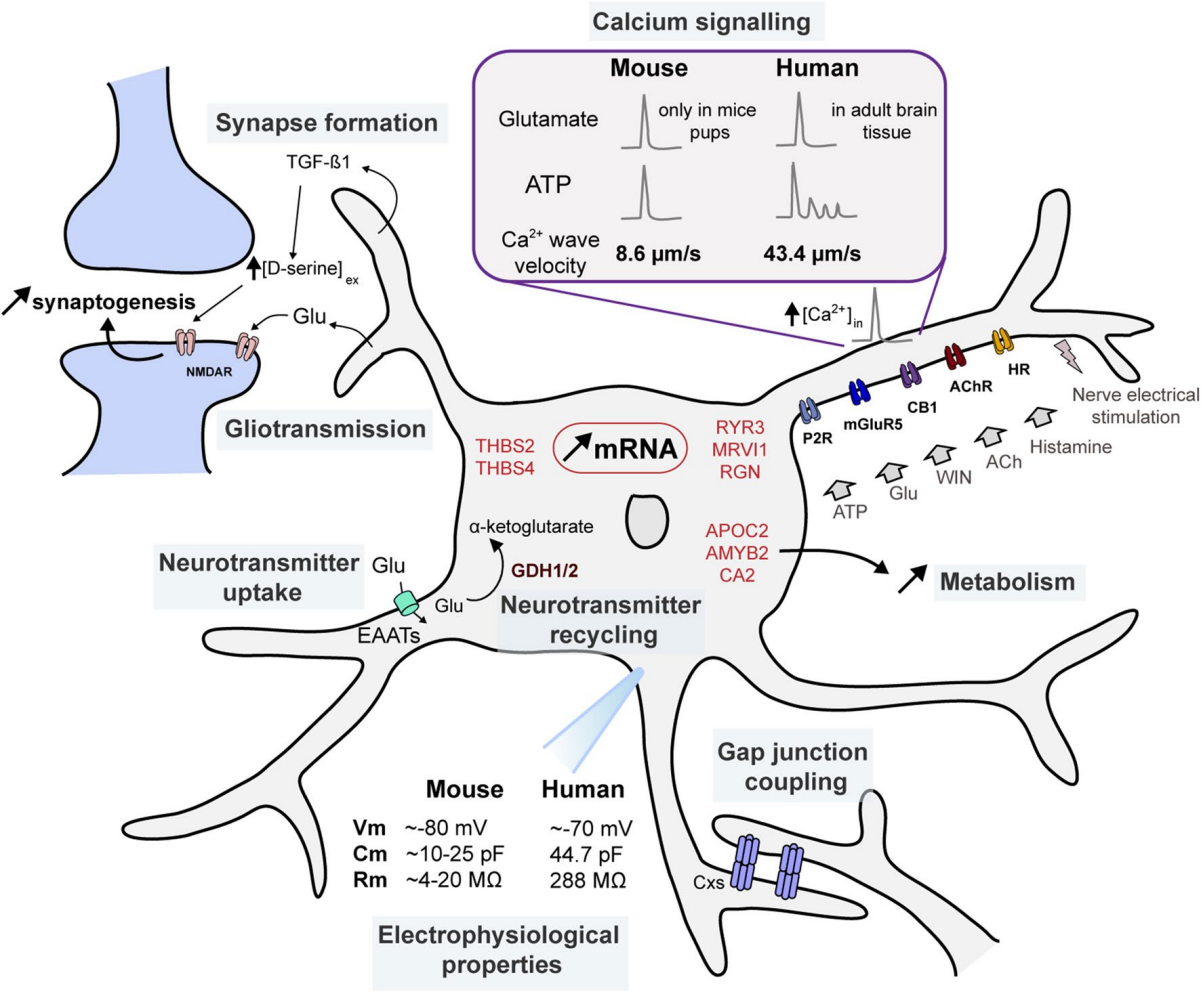
Genetic and functional specificities of human astrocytes. Schematic representation of genetic and functional specificities of human astrocytes in synapse formation, Ca^2+^ signalling, electrophysiological properties, gliotransmission, gap junction coupling, neurotransmitter uptake and recycling, and metabolism. mRNAs with higher expression in human astrocytes are indicated in *red. ACh* acetylcholine, *AChR* acetylcholine, *AMYB2* amylase α 2B, *APOC2* apolypoprotein C-2, *CA2* carbonic anhydrase 2, *CB1* cannabinoid receptor 1, *Cm* membrane capacitance, *Cxs* connexins, *EAATs* excitatory amino acid transporter, *GDH1/2* glutamate dehydrogenase 1/2, *Glu* glutamate, *HR* Histamine receptor, *mGluR5* metabotropic glutamate receptor 5, *MRVI1 (or IRAG)* inositol 1,4,5-triphosphate receptor-associated cGMP kinase substrate, *NMDAR* NMDA receptor, *P2R* purinergic receptor, *RGN* regucalcin, *Rm* membrane resistance, *RyR* ryanodine receptor, *THBS* thrombospondins, *Vm* membrane potential

Data from electrophysiological recordings, Ca^2+^ imaging, pharmacological and molecular studies have revealed numerous functions for human astrocytes that are similar to those attributed to rodent astrocytes. First, human astrocytes promote neuronal survival in the same way as in rodents (Zhang et al.  2016). Beyond promoting neuronal survival, they also contribute to synapse formation and synaptosomes engulfment, a function also ascribed to mouse astrocytes (Zhang et al. 2016). Going a step further, Diniz et al. (2012) investigated the mechanisms behind the assistance of astrocytes in synaptogenesis in both mouse and healthy human tissue resected from patients who had undergone surgical treatment of temporal lobe epilepsy (TLE). They found that the growth factor TGF-ß1 secreted by human astrocytes promoted synapse formation through a D-serine-mediated process that involved its NMDA receptor co-agonist role (Fig. 2). Strikingly, human astrocytes display enhanced efficiency in synaptogenesis activity and rely more heavily on TGF-ß1 signalling to ensure this function compared to mouse astrocytes (Diniz et al. 2012). An additional mechanism promoting synapse formation could involve thrombospondin secretion by astrocyte, since, as described earlier, THBS2 and THBS4 mRNAs were seen to be increased compared to non-human primates (Cáceres et al. 2007).

### Electrophysiological properties

The few available electrophysiological data on human astrocytes were extracted from human brain tissue surrounding astrocytomas, and tissue without significant histopathological hippocampal alterations from TLE patients (Picker et al. 1981; Bordey and Sontheimer 1998; Hinterkeuser et al. 2000). The resting membrane potential was found to average −63.9 mV to −70 mV, compared to ~ −80 mV in rodent astrocytes (Fig. 2). Here, it is important to note that the aforementioned studies did not account for the liquid junction potential, which might explain the difference with the resting membrane potential measured in rodents. In addition, the experiments were performed at room temperature, which might also affect cell membrane potential as well as input resistance. The capacitance of human astrocytes was also reported to be larger than the one of rodents (44.7 pF vs. ~10–25 pF), reflecting the bigger size of soma and proximal processes. Similarly, the membrane resistance was observed to be highly increased compared to rodent astrocytes (288 MΩ vs. ~4–20 MΩ), indicating a longer length constant that will allow current to travel further. While this could be seen as an evolutionary adaptation to the larger size of the astrocytes (Bordey and Sontheimer 1998; Dallérac et al. 2013), this observation seems counterintuitive, as a smaller membrane resistance might be expected from a larger, more capacitive cell.

### Gap junction-mediated intercellular coupling

Intercellular coupling through gap junction (GJ) channels formed by connexin 30 and 43 (Cx30 and Cx43), the GJ subunits in astrocytes, is a well-established astroglial function in the rodent. It allows the exchange of ions, metabolites, and neuromodulators, mediating signalling at the network scale (Pannasch and Rouach 2013). Expression and functions of the connexins enabling such intercellular communication have been extensively investigated in the human pathological—mostly epileptic and tumoural—brain (Lee et al. 1995; Aronica et al. 2001; Fonseca et al. 2002; Pu et al. 2004; Collignon et al. 2006; Caltabiano et al. 2010; Bedner et al. 2015). Therefore, the physiological characteristics of these proteins have to be inferred from the so-called control tissues—post-mortem or with few pathological hallmarks—used as a point of reference for the modulation of protein expression and function in the aforementioned studies. In particular, Cx43 was detected at the protein level, but there have been no report regarding Cx30 expression in the human brain (Aronica et al. 2001; Fonseca et al. 2002; Collignon et al. 2006) (Fig. 2). Second, it appears that there exist functional GJs in human astrocytes. Cultured astrocytes from normal tissue in patients with intractable mesial TLE (MTLE) were indeed found to display GJ coupling, as shown by fluorescence recovery after photobleaching (Lee et al. 1995). In tissues from non-sclerotic MTLE patients, biocytin-filling experiments also revealed GJ-coupled astrocytes (Bedner et al. 2015). Whether these GJs are composed of Cx43, or other connexin isoforms, remains to be determined.

### Neurotransmitter uptake and recycling

In the rodent brain, there is ample evidence for astrocytes mediating the rapid removal of neurotransmitter from the extracellular space, both to maintain spatial and temporal encoding of synaptic transmission, and to avoid excitotoxicity. This uptake occurs through different transporters such as GLT-1 and EAATs (allowing glutamate uptake) and GAT-1 (enabling GABA uptake) (Allen 2014). Immunohistochemical analysis of EAAT1 and EAAT2 glutamate transporters in the human post-mortem brain revealed that they both colocalise with astrocytes, and interestingly, most astrocytes were positive for either EAAT1 or EAAT2, indicating sub-categories of human astrocytes. This might relate to their morphological diversity (Banner et al. 2002). These glutamate transporters, beyond being expressed, appear to be functional: transient inward currents activated at negative voltages were detected by outside-out patch on astrocytes from non-sclerotic MTLE patients. These currents were blocked by DL-TBOA, a selective EAAT inhibitor, but not by NBQX, an AMPA receptor antagonist, indicating glutamate uptake through EAATs (Bedner et al. 2015) (Fig. 2). In an independent study, elevated glutamate concentrations—of up to 50 μM—in human fœtal astrocyte cultures, were found to stimulate glutamate uptake, as well as the increased membrane expression of EAAT1. Application of TBOA reversed the effect of glutamate application, indicating, again, the involvement of EAAT in the glutamate uptake (Gegelashvili et al. 2007). Data on properties of GABA uptake by astrocytes in the physiological human brain, however, remain sparse. A study looking at the effect of various antiepileptic agents on transport of GABA in cultured astrocytes from foetal tissue indicates that astrocyte-mediated GABA recapture does occur at early stages of development (Fraser et al. 1999).

Once re-uptaken, neurotransmitters are recycled and can be metabolised to support energy production (Bouzier-Sore and Pellerin 2013). In particular, the first step of glutamate metabolism, to enter in the TCA cycle, involves the enzyme glutamate dehydrogenase (GDH). Interestingly, while most mammals only express GDH1, humans express two GDH isoforms, GDH1 and 2 (Fig. 2). For instance, no mouse orthologue of GLUD2, the gene coding for GDH2, was found (Zhang et al. 2016). The exclusive expression of the enzyme in the human brain might signify an enhanced glutamate oxidation and more efficient TCA cycle during processes with high energy costs such as sustained glutamatergic activity. To test this hypothesis, Nissen et al. (2017) analysed glutamate uptake and oxidative metabolism in cultures of transgenic mice expressing the human GDH2 enzyme: they found that both were enhanced, especially during sustained activity and aglycemia. Equally interestingly, expression of the human GDH2 induced an increase of the use of branched-chain amino acids during aglycemia and a decrease in utilisation of oxidative glucose metabolism. This suggests that GDH2 enables an enhanced resistance to the extreme metabolic demands that might stem from intense excitatory neurotransmission and/or to restricted glucose supply (Nissen et al. 2017).

### Calcium signalling

Mirroring the increased expression of genes encoding Ca^2+^ signalling-related proteins in human astrocytes discovered in the transcriptome analysis by Zhang et al. (2016), Ca^2+^ transients in astrocytes were detected in human brain slices and astrocyte cultures by different groups. Navarrete et al. (2013) reported spontaneous Ca^2+^ rises in cortical and hippocampal astrocytes of tissues resected from patients undergoing surgery for drug-resistant TLE. These signals were independent of action potential-mediated neuronal activity, as shown by their TTX-insensitivity. Ca^2+^ elevations were also triggered by neurotransmitters such as glutamate, ATP, and WIN, a CB1 receptor agonist, as well as by nerve electrical stimulation (Navarrete et al. 2013). In addition, in human foetal astrocytes co-cultured with neurons, Ca^2+^ transients can be induced by other neuroactive molecules such as ADP, histamine or acetylcholine (Fu et al. 2013) (Fig. 2). Zhang et al. (2016) also reported specific Ca^2+^ responses in human astrocytes (Fig. 2): application of glutamate to mature astrocyte cultures induced a sharp intracellular Ca^2+^ increase in a dose-dependent manner, through the activation of metabotropic glutamate receptor 5 (mGluR5) (Zhang et al. 2016). In the mouse, this phenomenon has been seen to occur solely in astrocytes from mice pups (Sun et al. 2013), concurring with the high expression of mGluR5 under postnatal week 3 and its disappearance after this point. Notably, mGluR5 expression in human mature astrocytes has been also found to be low (Zhang et al. 2016), suggesting that mGluR5s in human astrocytes somehow perform better in the detection of synaptic activity or that other pathways mediating Ca^2+^ signalling are involved. Additionally, application of ATP in the same experimental conditions also triggered a Ca^2+^ response in astrocytes, with a different asynchronous and longer temporal pattern compared to the one elicited by either glutamate or ATP application in rat astrocytic cultures (Shigetomi et al. 2010). Notably, Navarrete et al. (2013) did not find such pronounced transmitter-specific Ca^2+^ dynamics, possibly owing to different systems in which they were studied (i.e. cultures vs. slices). It remains to be determined whether mouse astrocyte Ca^2+^ signalling displays the same heterogeneity depending on the neurotransmitter eliciting the response. One could hypothesise that the diversity in temporal kinetics might increase the information content and provide a code for the astrocyte to sense neuronal activity and to respond accordingly. Consistent with this finding, comparing astrocyte Ca^2+^ kinetics in acute slices of human brain tissue surgically resected from patients with different cerebral pathologies with that of mouse brain acute slices revealed a striking difference in Ca^2+^ wave transmission velocity (Oberheim et al. 2009). Human astrocyte Ca^2+^ waves propagated with an average velocity of 43.4 µm/s while the murine counterpart only reached 8.6 µm/s (Fig. 2). Increase in speed of propagation might come as a direct consequence of the increased size of astrocytes, and hence enhanced distance to travel. In mouse astrocytes, recent findings indicate compartmentalisation of Ca^2+^ transients within specific subcellular domains (soma vs. processes) to provide local patterns of Ca^2+^ signals occurring in a relatively independent manner (Araque et al. 2014; Rusakov et al. 2014; Volterra et al. 2014). Given that human astrocytes display a much larger size than their rodent counterparts, the relative independence of their subcellular domains may be even more prominent. Indeed, the volume of human astrocytes was reported to be ~16.5 times bigger than the ones of rodents, and might, therefore, ensure the regulation of some hundred thousand synapses (Oberheim et al. 2009). Because of the large range of direction (potentiation vs. depression) and scale (synapse vs. network) of astrocytic influence on neuronal activity, at least in the rodent brain, a compartmentalisation mechanism seems likely. The combined use of the GCaMP technology and organotypic human brain slices could prove useful to bring these finer, more local Ca^2+^ events to light. Indeed, Fluo-4, used in the studies presented, only allows visualisation and quantification of Ca^2+^ signals in somatic and main, proximal processes, and might conceal Ca^2+^ fluctuations in fine processes.

These reports provide invaluable information regarding Ca^2+^ signalling dynamics, but do not address the sources and targets of intracellular Ca^2+^. In the rodent, IP3-mediated release of Ca^2+^ from endoplasmic reticulum through binding to IP3Rs has been the subject of much attention in the recent years, and particularly IP3R2, owing to its predominant expression in astrocytes compared to neurons (Zhang et al. 2016). KO studies have suggested that, first, it modulates mainly somatic Ca^2+^ transients (Srinivasan et al. 2015), and second, that it does not regulate neuronal excitability, synaptic transmission and plasticity, neurovascular coupling or behaviour (Petravicz et al. 2008, 2014; Agulhon et al. 2010; Nizar et al. 2013; Bonder and McCarthy 2014). Alternative mechanisms to induce Ca^2+^ rises have been proposed, such as other IP3Rs or transient receptor potential cation channels A1 (TRPA1) (Shigetomi et al. 2011; Sherwood et al. 2017). As discussed, the emerging view that Ca^2+^ signalling might be compartmentalised suggests that Ca^2+^ elevations may be triggered through different pathways in the soma, branches and processes (Bazargani and Attwell 2016). To date, such characterisation in human astrocytes remains sparse. In human astrocytes, all three IP3Rs and TRPA1 appear to be expressed (Zhang et al. 2016), but functional investigation of their role in astrocytic Ca^2+^ signalling is still largely lacking. However, an electron microscopy study of human temporal lobe tissue did reveal the presence of all three isoforms of IP3Rs in secretory granules that might act as intracellular Ca^2+^ stores. Indeed, on these large dense core vesicules were found chromogranins A and B and secretogranin II, which are high-capacity, low-affinity Ca^2+^ storage proteins (Hur et al. 2010). A notable limitation to the investigation of the involvement of given proteins in the generation of these Ca^2+^ events is the difficulty to genetically engineer human tissue, a tool used extensively in the mouse model.

### Gliotransmission

Human astrocytes are able to sustain complex Ca^2+^ signals. The natural next question is: can the downstream signals of Ca^2+^ fluctuations lead to gliotransmission, as it does in the rodent (Parri et al. 2001; Perea and Araque 2005; Haydon and Carmignoto 2006; Navarrete and Araque 2008; Shigetomi et al. 2008; Bardoni et al. 2010)? Navarrete et al. (2013) provides data indicating that it does, at least in tissue resected from patients undergoing surgery for drug-resistant TLE. Indeed, slow inward currents, mediated by NMDA receptor activation, were electrophysiologically detected in neurons, and their frequency was increased after the application of ATP that induced Ca^2+^ rises in astrocytes (Navarrete et al. 2013) (Fig. 2).

### Insights from human-mouse chimeras

As access to brain tissue resected during surgical procedures remains limited and its usage only allows ex vivo studies, an alternative tool has been developed by Han et al. (2013) to assess human astrocyte functions in an integrated manner: engraftment of human glial progenitor cells (GPCs) into the forebrain of neonatal immunodeficient mice (Han et al. 2013). These GPCs persisted throughout development and maturation, were able to migrate throughout the cortex and hippocampus, and to differentiate into astrocytes that displayed hominid features, namely large size, complex morphology and fast Ca^2+^ propagation. Interestingly, these cells incorporated into the network of mouse astrocytes, coupling through GJs with host astrocytes and forming endfeet on host blood vessels. Han and colleagues then investigated the functional impact of engraftment human GPCs in mouse brains on neuronal networks and cognition. First, it was reported that field excitatory postsynaptic potentials were higher in the grafted dentate gyrus of the hippocampus compared to ungrafted and allografted littermate controls, revealing an increase in the basal level of excitatory synaptic transmission. Next, high frequency stimulation in hippocampal slices was shown to induce a stronger and longer-lasting long-term potentiation (LTP), which was mediated by a postsynaptic mechanism. How do human astrocytes enhance LTP through a postsynaptic process? While the implication of adenosine or D-serine was ruled out, a mechanism involving the release of cytokine TNFα to promote the insertion of AMPA receptors at the neuronal membrane was proposed. Enhanced plasticity reflected by increased LTP could affect human cognition by, for example, facilitating network reorganisation as a response to change in environmental input. In a second part of the study, a series of learning tests have been used to ask whether enhanced LTP reflected better learning abilities. Strikingly, performance in contextual fear conditioning, Barnes Maze, Object-Location Memory Task—all hippocampus-dependent tasks—and auditory fear conditioning, were all enhanced in mice engrafted with human GPCs, corroborating ex vivo experiments. While this shows that engraftment of human GPCs improves mice synaptic transmission, plasticity and cognitive functions, the cellular mechanisms involved in the phenomenon remain unclear. As Zhang and Barres (2013) astutely observed, a notable limitation of this work is that it is difficult to discriminate between the role of GPCs and that of astrocytes in the modification of neuronal networks and cognitive properties observed in the engrafted mice, since both were present. Moreover, as described earlier, human astrocytes display a vast heterogeneity depending on the regions, and it is difficult to ascertain that the astrocytes generated were location-appropriate (Zhang and Barres 2013). Nevertheless, Han and colleagues provided the first evidence that human GPCs enhance cognitive functions through an as-yet-unknown mechanism.

## Conclusion

Major discoveries regarding astrocytes’ functional importance relied on studies performed mostly on the rodent animal model, providing a strong but limited foundation for overall understanding of the role of astrocytes in physiopathology. Recent technical and methodological advances allowing the investigation of astrocyte behaviour in the human brain have partially confirmed findings in rodents, but have also unveiled discrepancies, warranting the necessity for verification on human samples. Strikingly, morphological, genomic and functional studies revealed notable characteristics specific to human astrocytes, attributing them properties that might supposedly sustain more complex information processing. Specifically, human astrocytes were discovered to display a remarkable morphological diversity according to cortical layers, which indicates a high degree of specialisation and, therefore, enhanced performance at more specific tasks, such as long-distance communication (interlaminar astroglia), information integration (protoplasmic astroglia), and metabolic support (fibrous astroglia). Importantly, both genomic and functional studies corroborated to show a specific relevance of intracellular Ca^2+^ concentration fluctuations, revealing a high expression of genes coding for proteins involved in Ca^2+^ signalling (RYR3, MRVI1 and RGN), more diverse patterns of Ca^2+^ signalling and faster propagation of Ca^2+^ waves. In light of findings in rodents establishing Ca^2+^ transients in astrocytes as a potential signalling system interacting with the neuronal signalling system (Bazargani and Attwell 2015), enhanced Ca^2+^ signalling may provide the basis for a more sophisticated neuron-astroglia dialogue with faster processing of more complex calculations. As one would expect, an increased energy supply is required to sustain this heightened computational power. And indeed, APOC2, AMY2B and CA2, genes coding for proteins involved in metabolism, were seen to be enriched in human astrocytes. The possibility that astrocytes might have an even more central role in neuronal and synaptic functions, as suggested by these observations, indicates an equivalent involvement in human cerebral pathology. Certainly, astrocyte dysfunction is a characteristic of many CNS diseases such as epilepsy, brain tumours and Alzheimer’s disease. To efficiently investigate astrocytic dysregulation in these pathologies, it is crucial to understand the functional significance of the species-specific properties of normally-operating human astrocytes.
